# When the lights go out: the evolutionary fate of free‐living colorless green algae

**DOI:** 10.1111/nph.13279

**Published:** 2015-01-26

**Authors:** Francisco Figueroa‐Martinez, Aurora M. Nedelcu, David R. Smith, Adrian Reyes‐Prieto

**Affiliations:** ^1^ Biology DepartmentUniversity of New BrunswickFrederictonNBE3B 5A3Canada; ^2^ Biology DepartmentUniversity of Western OntarioLondonONN6A 5B7Canada; ^3^ Integrated Microbiology ProgramCanadian Institute for Advanced ResearchTorontoON M5G 1Z8Canada

**Keywords:** colorless algae, loss of photosynthesis, mixotrophism, *Polytoma*, *Polytomella*

## Abstract

The endosymbiotic origin of plastids was a launching point for eukaryotic evolution. The autotrophic abilities bestowed by plastids are responsible for much of the eukaryotic diversity we observe today. But despite its many advantages, photosynthesis has been lost numerous times and in disparate lineages throughout eukaryote evolution. For example, among green algae, several groups have lost photosynthesis independently and in response to different selective pressures; these include the parasitic/pathogenic trebouxiophyte genera *Helicosporidium* and *Prototheca*, and the free‐living chlamydomonadalean genera *Polytomella* and *Polytoma*. Here, we examine the published data on colorless green algae and argue that investigations into the different evolutionary routes leading to their current nonphotosynthetic lifestyles provide exceptional opportunities to understand the ecological and genomic factors involved in the loss of photosynthesis.

## Introduction

### The rise, spread, and loss of eukaryotic photosynthesis

**Figure 1:**
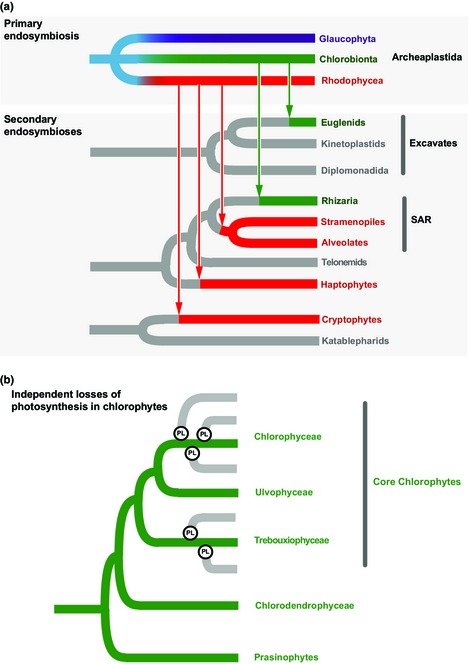
Schematic trees illustrating the distribution of plastid‐bearing lineages among the major Eukaryote ‘supergroups’. (a) The primary endosymbiotic origin of plastids presumably gave rise to the photosynthetic common ancestor of the three Archaeplastida groups: Chlorobionta (land plants and green algae), Rhodophycea (red algae) and Glaucophyta. After the establishment of the primary plastids, different eukaryote groups acquired plastids via independent secondary endosymbioses. Secondary plastids derived from red algal endosymbionts (red lines) are present in the alveolates (chromerids, dinoflagellates and apicomplexans), stramenopiles (diatoms and brown algae), haptophytes, and cryptophytes. The presence of secondary plastids of green algal origin (green lines) is recognized in some rhizarians (chlorarachniophytes) and excavates (euglenids). In addition to their ancestral red algal plastids, dinoflagellates are well known by the multiple independent cases of plastid replacements and even the presence of plastids of tertiary origin (i.e. recruited from algae with secondary plastids; not shown). The position of the vertical arrows does not reflect the age of the secondary endosymbiotic events. (b) The schematic phylogenetic tree of the major core chlorophyte classes (Chlorophyceae, Ulvophyceae and Trebouxiophyceae) illustrates the diverse colorless (gray lines) lineages described: the orders Chlorellales (Trebouxiophyceae) and Chlamydomonadales (Chlorophyceae). Phylogenetic evidence (Figs 2, 3) demonstrates that the loss of photosynthesis has occurred at least three different times in Chlamydomonadales and possibly two times in Chlorellales. However, the different unicellular colorless algal lineages have different ecological‐evolutionary histories and have followed distinct patterns. The known colorless Chlorellales have evolved via pathogenic/parasitic routes, whereas the nonphotosynthetic Chlamydomonadales presumably have evolved in free‐living contexts. PL, plastid loss; SAR, Stramenopiles, Alveolata, and Rhizaria.

Approximately 1.5 Gyr ago (Yoon *et al*., 2004), eukaryotes acquired photosynthetic capabilities by establishing an endosymbiotic relationship with a cyanobacterium – an event that ultimately gave rise to the plastids of all photosynthetic eukaryotic lineages (Keeling, 2010). It is widely accepted that the plastids of glaucophytes, red algae, green algae, and land plants – which together form the Archaeplastida supergroup – evolved directly from a common cyanobacterial ancestor (i.e. ‘primary’ plastids; Fig. 1). The plastids of other eukaryotic lineages, however, were acquired through more recent eukaryote–eukaryote endosymbioses, and are known as ‘secondary’ or ‘tertiary’ plastids (Keeling, 2010). For instance, the plastids of stramenopile algae (e.g. diatom, brown, and golden algae), various alveolates (e.g. dinoflagellates and chromerids), haptophytes, and cryptophytes arose from secondary endosymbioses with red algae. Conversely, the plastids of chlorarachniophytes (Rhizaria) and photosynthetic euglenids (euglenophytes; Excavata) evolved from independent secondary endosymbioses with green algae (Fig. 1a) (Keeling, 2010).

Understanding plastid evolution becomes even more complex when considering that many algal lineages have lost photosynthesis independently (Fig. 1a) (Keeling, 2010). Photosynthesis was also lost in land plants, as exemplified by the parasitic, nonphotosynthetic land plants *Epifagus virginiana*,* Orobanche minor*,* Rafflesia lagascae*, and different *Cuscuta* isolates (Wicke *et al*., 2013), which obtain water and other nutrients directly from the vascular system of the parasitized host. Most eukaryote lineages that have lost photosynthesis still retain colorless (lacking photosynthetic pigments) plastids, which continue to perform crucial nonphotosynthetic metabolic functions (Wicke *et al*., 2013).

Unraveling the events that resulted in the astonishing diversity of photosynthetic eukaryotes and their nonphotosynthetic close relatives requires a deep understanding of the adaptive and nonadaptive forces and genomic consequences associated with the gain and loss of photosynthesis. Here, we examine recently published data on colorless chlorophyte green algae and argue that investigations into the different evolutionary routes leading to their nonphotosynthetic lifestyles provide exceptional opportunities to understand the ecological and genomic factors involved in the loss of photosynthesis.

### Mixotrophism: from heterotrophism to photoautotrophism and back

Heterotrophism and autotrophism take advantage of different carbon sources (organic vs inorganic), and organisms evolved various ways to utilize one or the other. However, ‘mixotrophic’ organisms can make use of both inorganic (via photoautotrophism) and organic (via chemoheterotrophism) carbon sources; the latter involves prey consumption through phagocytosis (phago‐mixotrophism), endocytosis, or the intake of small organic compounds via osmosis (osmo‐mixotrophism). Whether or not photoautotrophy or chemoheterotrophy is the main form of nutrient assimilation varies among mixotrophic organisms and depends on the availability of light and organic compounds in the environment (Troost *et al*., 2005). Consequently, mixotrophs should outcompete obligate photoautotrophs in environments where light or low inorganic supplies limit the photosynthetic activity, and have an advantage over strict heterotrophs when prey or dissolved organic compounds are scarce (Tittel *et al*., 2003). Despite its apparent benefits, mixotrophy has a major drawback: it is costly to maintain the molecular machinery needed for both trophic strategies. It is estimated that mixotrophic protists spend five times more energy and nutrient allocation on maintaining the photosynthetic apparatus than on heterotrophic abilities (Raven, 1997). This implies that under certain conditions, such as when the energy costs of maintaining the photosynthetic apparatus outweigh the benefits of its products, the selective pressures on preserving photoautotrophic machinery can be relaxed and the loss of photosynthesis – even under favorable light conditions – can be an ecological advantage (De Castro *et al*., 2009).

Indeed, the presence of numerous plastid‐harboring nonphotosynthetic groups demonstrates that photosynthesis is dispensable under certain conditions, and that the loss of photosynthesis is not uncommon among mixotrophic algae (Stoecker, 1998). Extant colorless algal lineages have either phagotrophic or osmotrophic lifestyles, and this is generally a reflection of the heterotrophic strategy employed by their mixotrophic relatives. For example, phagotrophic colorless algae can be found among dinoflagellates, stramenopiles and cryptophytes; this lifestyle is consistent with the presence of phagotrophism in their close mixotrophic relatives. Other colorless algae, such as the chlorophyte green algae *Helicosporidium*,* Prototheca*,* Polytoma*, and *Polytomella*, are closely related to osmo‐mixotrophic chlorophytes and adopted an osmotrophic strategy, where the source of dissolved organic matter can be either a host (in the case of pathogenic/parasitic species) or the environment (in free‐living species). Interestingly, although there are no reported cases of phagotrophic colorless green algae, a few examples of phago‐mixotrophic prasinophytes are known (Maruyama & Kim, 2013).

## Chlorophyte green algae as models in which to study the loss of photosynthesis

The Chlorophyta comprises a diverse assemblage of green algae traditionally classified into Chlorophyceae, Trebouxiophyceae, Ulvophyceae and Prasinophyceae. The loss of photosynthesis has occurred several independent times among chlorophytes: at least twice in the order Chlorellales (Trebouxiophyceae) and at least three times in the order Chlamydomonadales (Chlorophyceae) (Fig. 1b). These unicellular nonphotosynthetic algae are particularly interesting because they each have distinct and disparate ecological and evolutionary histories leading to their obligate heterotrophic lifestyles: colorless species from the order Chlorellales evolved as opportunistic parasites/pathogens, whereas the colorless Chlamydomonadales lost photosynthesis as free‐living organisms.

### Parasitism and the loss of photosynthesis in Chlorellales (Trebouxiophyceae)

The genera *Prototheca* and *Helicosporidium* (Trebouxiophyceae, Chlorellales) include unicellular nonflagellated parasites/pathogens that still retain vestigial plastids. Members of the genus *Prototheca* are ubiquitous opportunistic animal pathogens that can be found in diverse habitats, such as soil detritus, fresh and brackish water, and plant‐ and animal‐derived foods for human consumption. *Prototheca* is the causative agent of protothecosis, a disease that develops after *Prototheca* comes in contact with skin wounds, causing cutaneous lesions, bursitis, and major systemic alterations in immunosuppressed hosts (Lass‐Flörl & Mayr, 2007). Protothecosis is rare in humans with only 160 cases documented in the medical literature between 1964 and 2011 (Todd *et al*., 2012). *Helicosporidium* infections are common in insects, mites, trematodes and cladocerans (Tartar, 2013). Recent reports indicate that *Helicosporidum* infections can affect between 10% and 70% of coleopteran populations (Yaman, 2008).

**Figure 2:**
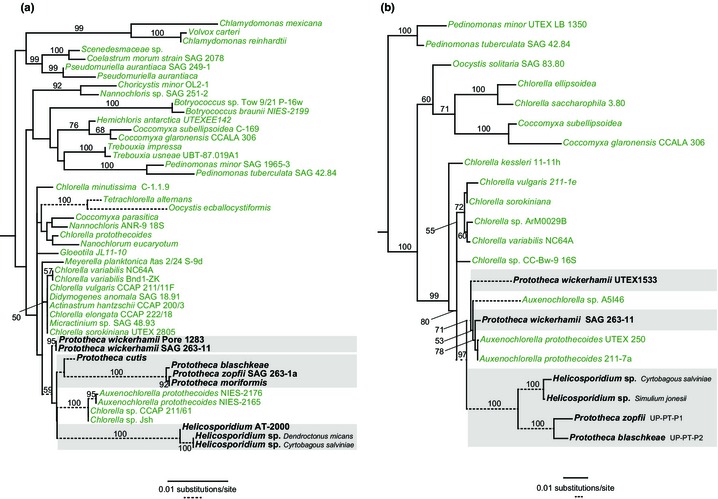
Maximum likelihood (ML) trees of photosynthetic and colorless Chlorellales. Maximum likelihood phylogenetic analyses of (a) nuclear 18S rRNA and (b) plastid 16S rRNA gene sequences are shown. Sequences were aligned with mafft v7 (Katoh & Standley, 2013) and manually refined using se‐al v2.0a11 (http://tree.bio.ed.ac.uk/software/seal/). ML trees were estimated using raxml considering the GTR + G model, which was identified as the best‐fit substitution model according to the Akaike information criterion (AIC) criterion of modeltest 2.1.4. Branch support was assessed with 500 bootstrap replicates. Numbers near nodes indicate ML bootstrap support (only values > 50% are shown). Branch lengths are proportional to the number of substitutions per site indicated by the scale bars. To accommodate long branches of certain taxa, two different branch scales (solid and dotted lines) are displayed. Gray boxes highlight colorless taxa.

The loss of photosynthesis probably occurred in the ancestors of *Prototheca* and *Helicosporidium* during their shift from mixotrophy to parasitism (Pombert *et al*., 2014). It is unclear, however, if these two closely related genera lost their photosynthetic abilities independently. Recent phylogenetic analyses of nuclear 18S rRNA and β‐tubulin data have shown that some *Prototheca wickerhamii* isolates are more closely related to photosynthetic taxa (e.g. *Chlorella* spp.) than to other *Prototheca* species (Mancera *et al*., 2012). Our maximum likelihood (ML) analyses of nuclear 18S rRNA (Fig. 2a) and plastid 16S rRNA (Fig. 2b) sequences from various trebouxiophytes depict *P. wickerhamii* SAG 263‐11 as a nonsister lineage to the other *Prototheca* and *Helicosporidium* species. These data suggest that the loss of photosynthesis has occurred at least twice in the evolution of parasitic/pathogenic Chlorellales (Fig. 2). Moreover, the mixotrophic capabilities of various *Chlorella* species (Lee *et al*., 1996), which are able to use different organic compounds (e.g. glucose, glycerol, ethanol, acetate, and butyrate) as carbon sources, imply that nonphotosynthetic Chlorellales probably evolved from commensals (e.g. saprophytes; similar to *Prototheca* species living in animal integumentary tissues) that ultimately harnessed their heterotrophic abilities to invade novel ecological niches.

### Multiples cases of photosynthesis loss in free‐living colorless Chlamydomonadales (Chlorophyceae)

**Figure 3:**
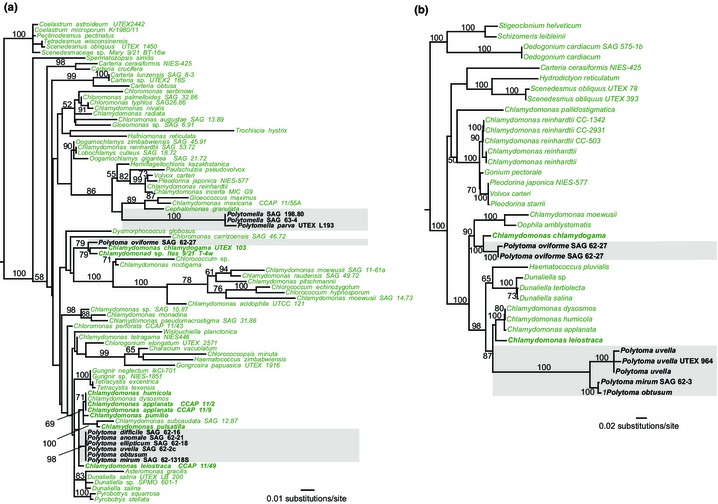
Maximum likelihood (ML) trees of photosynthetic and colorless Chlamydomonadales. ML phylogenetic analyses of (a) nuclear 18S rRNA and (b) plastid 16S rRNA gene sequences are shown. Nucleotide sequences were aligned with mafft v7 (Katoh & Standley, 2013) and manually refined. ML trees were estimated with raxml considering the GTR + G nucleotide substitution model. Branch support was assessed with 500 bootstrap replicates. Numbers near nodes indicate ML bootstrap support (only values > 50% are shown). Branch lengths are proportional to the number of substitutions per site indicated by the scale bars. Gray boxes highlight colorless taxa.

*Polytoma* and *Polytomella* are two nonphotosynthetic genera that belong to the Chlamydomonadales (Chlorophyceae) (Fig. 3) (Nakada *et al*., 2008). Both lineages consist of free‐living, flagellated heterotrophs that live in fresh water and have vestigial plastids with notable morphological similarities to the colorless plastids of certain *Chlamydomonas reinhardtii* nonphotosynthetic mutants (Inwood *et al*., 2008). *Polytomella* and *Polytoma* species can use various compounds as carbon sources, including organic acids (pyruvate, acetate, succinate and butyrate), alcohols (ethanol and butanol) and monosaccharides (glucose and glyceraldehyde) (Links *et al*., 1961).

Phylogenetic analyses using different molecular markers indicate that *Polytomella* is a monophyletic group, whereas the *Polytoma* genus is polyphyletic and comprises at least two independent lineages (Nedelcu, 2001; Nakada *et al*., 2008). Our ML phylogenetic analysis of nuclear 18S rRNA sequences (Fig. 3a) indicates that certain *Polytoma* species (*Polytoma uvella*,* Polytoma mirum*,* Polytoma obtusum*, and others) are closely related to the osmo‐mixotrophic *Chlamydomonas leiostraca* and *Chlamydomonas humicola* as well as to other photosynthetic species, including *Chlamydomonas applanata*,* Chlamyomonas pulsatilla*, and *Chlamydomonas pumilio*. The colorless *Polytoma oviforme* (SAG 62‐27), however, branches independently of other *Polytoma* species and forms a sister lineage to the photosynthetic *Chamydomonas chlamydogama* and Chlamydomonad sp. Itas 9/21 T‐4w (Fig. 3a). Although the position of *Polytomella* species is not well resolved in our 18S rRNA analysis, they do form a monophyletic group closely related to the photosynthetic species *C. reinhardtii* and *Volvox carteri* (Smith & Lee, 2014). The ML analysis of the plastid 16S rRNA (Fig 3b) is consistent with the 18S rRNA ML trees, placing (1) *P. uvella*,* P. obtusum*, and *P. mirum* close to the photosynthetic *C. leiostraca*,* C. humicola* and *C. applanata*, and (2) *P. oviforme* as sister to *C. chlamydogama* (100% BS; note that *Polytomella* species were not included in the plastid tree because they have lost their plastid genomes (Smith & Lee, 2014)). Taken together, these phylogenetic analyses suggest that the loss of photosynthesis has occurred at least three times independently within the Chlamydomonadales.

### Loss of photosynthesis in Chlamydomonadales: insights from *Chlamydomonas reinhardtii* colorless mutants

*Chlamydomonas reinhardtii* and other species, such as *C. humicola* and *Chlamydomonas acidophila*, can grow in total darkness, heterotrophically, with acetate as the only carbon source. When *C. reinhardtii* is grown under mixotrophic and saturating light conditions, the use of acetate as a carbon source significantly inhibits photosynthetic metabolism without affecting the rate of cell growth. Even under optimal light conditions for photosynthesis, the number of carbon compounds derived from acetate consumption can replace up to 50% of the photoautotrophically fixed carbon (Heifetz *et al*., 2000), Thus, it is reasonable to hypothesize that, under particular environmental conditions (e.g. low inorganic nutrients and low light intensity), the photosynthetic machinery can become expendable in mixotrophic algae.

Studies of certain nonphotosynthetic *C. reinhardtii* mutants suggest that the loss of photosynthesis can emerge from single‐nucleotide mutations of genes involved in photopigment biosynthesis (e.g. phytoene or chlorophylls). For instance, the *C. reinhardtii* ‘white’ mutant *lts1‐204*, defective in phytoene synthase (an enzyme catalyzing the first step of carotenoid biosynthesis), and ‘yellow’ mutants, defective in Mg‐protoporphyrine IX methyltransferase (an enzyme involved in tetrapyrrole biosynthesis), produce stable phenotypes with no carotenoid pigments and a low chlorophyll concentration, respectively (McCarthy *et al*., 2004; Meinecke *et al*., 2010). Both nonphotosynthetic mutants can grow in the dark using acetate as a carbon source, but are unable to survive even under low light conditions. The lack of carotenoids in *C. reinhardtii* ‘white’ mutants affects the assembly of reaction centers and concomitantly the structure of the thylakoids, resulting in plastids with no stacked thylakoidal membranes similar to the colorless plastid of *Polytoma* (Inwood *et al*., 2008). The fact that these colorless mutants can proliferate in the dark using solely organic carbon sources (e.g. organic acids or monosaccharides) implies that mutations of this type would be nearly neutral in environments where photosynthesis is not critical for carbon assimilation, and offers an ecological scenario and a plausible explanation for the origin of free‐living heterotrophic colorless algae. Furthermore, the numerous *Chlamydomonas* ‘white’ and ‘yellow’ mutants are promising models for studying the physiological and genetic mechanism underlying this major trophic shift.

## Genomic consequences of the loss of photosynthesis: different routes, different endpoints?

The different selective pressures and lifestyles (e.g. parasitism vs free‐living) associated with the evolution of the colorless green algae discussed in the previous subsection are likely to be reflected in distinct genomic evolutionary processes in these lineages. For instance, parasitism is expected to result in genome reduction (e.g. large‐scale loss of genes) or compaction (e.g. shorter genes and intergenic regions; loss of introns). However, the recently sequenced nuclear genome of the parasite *Helicosporidium* sp. ATCC 50920 does not exhibit the large levels of reduction generally observed in nuclear genomes of other unicellular parasitic species (e.g. apicomplexans, microsporodians and ciliates (Corradi *et al*., 2010; Coyne *et al*., 2011; Heitlinger *et al*., 2014). Although the *Helicosporidium* genome shows some evidence of compaction, most of the cellular functions found in free‐living, photosynthetic Chlorellales are still present in *Helicosporidium*, with the exception of those involved in photosynthesis (Pombert *et al*., 2014). The lack of nuclear genome reduction in *Helicosporidium* possibly reflects a recent transition to a parasitic lifestyle and might also be associated with the ability of *Helicosporidium* to grow independently of its host in experimental cultures (Pombert *et al*., 2014).

### Plastid genomics of nonphotosynthetic chlorophytes

The plastid genomes (ptDNA) of chlorophytes have revealed interesting evolutionary patterns. Comparative analyses of diverse chlamydomonadalean species have shown that ptDNA size and amount of noncoding regions correlate positively with the level of cellular organization, whereby multicellular taxa or taxa with large cells have larger ptDNAs than unicellular species or those with small cells (Smith *et al*., 2013). Furthermore, the ptDNAs of photosynthetic Chlamydomonadales exhibit remarkable architectural diversity (Smith *et al*., 2013). For example, the 525‐kb ptDNA of *V. carteri*, which is *c*. 80% noncoding and contains *c*. 96 genes (protein‐coding genes, rRNAs and tRNAs), is among the largest plastid chromosomes sequenced thus far (Smith & Lee, 2010); the ptDNAs of the chlamydomonadaleans *Dunaliella salina* and *Pleodorina starrii* are also very large (both *c*. 270 kb) and contain 104 and 103 genes, respectively. The ptDNAs of Chlorellales also show a range of architectures: *Chlorella variabilis* (124 kb and *c*. 102 genes; HQ914635), *Chlorella sorokiniana* (109 kb and *c*. 100 genes; GenBank: KJ742376), *Chlorella vulgaris* (150 kb and *c*. 101 genes; AB001684), *Parachlorella kessleri* (*c*. 124 kb and *c*. 105 genes; FJ968741), *Oocystis solitaria* (incomplete ptDNA; 96 kb and 108 genes; FJ968739) and *Auxenochlorella protothecoides* (incomplete ptDNA; 84 kb and 99 genes; KC631634).

In contrast to the number of data available for photosynthetic chlorophyte lineages, little is known about the evolutionary patterns characterizing the plastid genomes of their nonphotosynthetic relatives. The loss of photosynthesis is typically associated with ptDNA reduction and the erosion of photosynthesis‐related genes (De Koning & Keeling, 2006). The ptDNAs of *Helicosporidium* and *Prototheca* species have shorter intergenic regions, fewer introns, and reduced coding capacities as compared with photosynthetic Chlorellales (De Koning & Keeling, 2006). For instance, the *Helicosporidium* sp. ATCC 50920 ptDNA is 37.5 kb with only 29 protein‐coding genes, none of which are associated with photosynthesis. Similarly, the available *c*. 55‐kb ptDNA sequence for *P. wickerhamii* encodes 18 proteins unrelated to photosynthesis (Tartar & Boucias, 2004). Although additional data are still needed to clarify whether or not *Prototheca* and *Helicosporidium* evolved independently or share a common nonphotosynthetic ancestor, it appears that evolution towards parasitism has produced similar plastid gene content in colorless parasitic green algae (De Koning & Keeling, 2006). In contrast, ptDNA data from free‐living, colorless chlamydomonadaleans are rather limited. Interestingly, a recent investigation of *Polytomella* species has demonstrated that, although the nonphotosynthetic plastids of these colorless algae are metabolically active, they have completely lost their genome, taking the process of genome reduction to the ultimate extreme (Smith & Lee, 2014). *Polytomella* is one of only two known examples of ptDNA loss (the other is the parasitic angiosperm *Rafflesia lagascae*; Molina *et al*., 2014). By contrast, *Polytoma* species do contain a ptDNA, but available information is restricted to 16S rRNA and few protein‐coding sequences.

### Comparative ‘omics’ and the plastid functions in colorless chlorophytes

As discussed above, phylogenetic, genomic, and physiological data all indicate that the widespread mixotrophic capabilities of unicellular green algae underlie the multiple ‘successful losses’ of photosynthesis. Central to understanding the evolution of colorless green algae is the identification of physiological (e.g. particular pathways) and genetic (e.g. key mutations) mechanisms involved in the ‘no‐return’ transition from a mixotrophic lifestyle to an obligate heterotrophic one, and the role of nonphotosynthetic plastids in colorless algae. Further comprehensive genomic and functional investigations are critical to a better understanding of how these major trophic shifts occurred several times independently in chlorophytes.

Nuclear genome sequencing of various photosynthetic chlamydomonadalean and trebouxiophyte algae has revealed a surprising amount of genomic architectural diversity. The nuclear genomes of the chlamydomonadalean *C. reinhardtii* (121 Mbp) (Merchant *et al*., 2007) and *V. carteri* (138 Mbp) (Prochnik *et al*., 2010) are more than twice the size and have one‐third more protein‐coding genes ( *c*. 14 800 vs *c*. 9800; see Table 1 for details) compared with those of the trebouxiophyte *C. variabilis* (46 Mb) (Blanc *et al*., 2010) and *Coccomyxa subellipsoidea* (48.8 Mbp) (Blanc *et al*., 2012) (Table 1). Moreover, the gene complements of these green algal species are considerably different. For instance, *C. subellipsoidea* shares only 65% of its gene collection with *C. variabilis* and 56% with *C. reinhardtii*; and only 53% of the *C. variabilis* genes have orthologs in the *C. reinhardtii* genome (Blanc *et al*., 2010, 2012).

**Table 1:** Comparison of nuclear genome data from several Chlamydomonadales and Chlorellales green algae

	*Chlre*	*Volca*	*Chlva*	*Cocsu*	*Helic*
Genome size (Mb)	121.0	138.0	46.0	48.8	17
Chromosome number	17	14	12	20	10
Number of predicted genes	15 143	14 520	9791	9851	6035
Exons per gene	8.3	7.0	7.3	7.0	2.3
Coding sequences (%)	16.7	18.0	29.0	n/a	n/a
Repeated sequences (%)	16.7	23.8	8.9	7.2	n/a

*Chlre*,* Chlamydomonas reinhardtii*;* Volca*,* Volvox carteri f. nagariensis*;* Chlva*,* Chlorella variabilis* NC64A; *Cocsu*,* Coccomyxa subellipsoidea* C‐169; *Helic*,* Helicosporidum* sp. ATCC 50920; n/a, data not available.

Understanding the functions of colorless plastids requires the identification of the nuclear‐encoded, plastid‐targeted proteins (i.e. the colorless plastid proteome). In photosynthetic lineages, proteins encoded in the ptDNA contribute very little to the overall plastid proteome, and the vast majority (95–98%) of proteins serving plastids are nuclear encoded (Keegstra & Cline, 1999). Proteomic studies and predictions indicate that the plastid proteome of green algae and land plants comprises between 2500 and 4000 proteins (Van Wijk & Baginsky, 2011). Considering 1400 (those experimentally verified) as the lower limit of nuclear‐encoded, plastid‐targeted proteins and 9800 as the overall nuclear gene complement identified in photosynthetic Chlorellales, it follows that *c*. 15% of the nuclear gene repertoire could be potentially affected by the loss of plastid functions.

However, information on the nuclear gene repertoire and proteome of colorless chlorophytes is quite limited. The sequencing of the *Helicosporidium* sp. ATCC 50920 nuclear genome (Pombert *et al*., 2014) has provided a strong foundation for comparative genomics between photosynthetic and colorless green algae. The nuclear genome of *Helicosporidium* (*c*. 17 Mbp) is almost two‐thirds smaller and encodes about one‐third fewer genes than the genomes available from photosynthetic Chlorellales. Moreover, *c*. 15% (882 sequences) of the *Helicosporidium* nuclear genes have no recognizable orthologs in *C. subellipsoidea* and *C. variabilis* (Pombert *et al*., 2014). The reduction in coding capacity of the *Helicosporidium* nuclear genome is mainly a result of the contraction of gene families involved in DNA packing, transcription, and protein translation and modification, rather than the loss of metabolic capabilities observed in genomes of other colorless parasites, such as apicomplexans (Abrahamsen *et al*., 2004) and some microsporidian species (Corradi *et al*., 2010). These findings suggest that genome reduction is an ongoing process in *Helicosporidium*, and one that primarily affects the light harvesting, photosynthetic electron transport chain, and carbon fixation pathways (Pombert *et al*., 2014). In addition to contractions, gene family expansions are also observed in *Helicosporidium*, including 14 genes encoding glycosyl hydrolases of the GH18 chitinase family; these expansions are probably associated with the evolution towards parasitism (Pombert *et al*., 2014).

Previous transcriptomic investigations of *Helicosporidium* sp. and *Prototheca wickerhamii* have suggested that the plastids of these colorless Chlorellales house critical biosynthetic pathways (Borza *et al*., 2005; Pombert *et al*., 2014). The *Helicosporidium* genome sequence has confirmed that most of the plastid enzymes participating in biosynthesis of aromatic and hydrophobic side‐chain amino acids, fatty acids, tetrapyrrole, and terpenoids are encoded in the nucleus. Most of the genes involved in the Calvin–Benson cycle, starch biosynthesis, the TIC/TOC (translocons at the inner and outer envelope membranes, respectively, of plastids) machinery (plastid protein import), and the general secretory (SEC) pathway (protein export) are also nuclear encoded. Although proteins for many plastid‐localized pathways are still encoded in the nuclear genome, nearly 44% of the plastid‐targeted proteins present in other viridiplants (green algae and land plants) have probably been lost from the *Helicosporidium* metabolic repertoire (Pombert *et al*., 2014). The presence of conserved plastid pathways in *Helicosporidium* and *Prototheca* indicates that these biochemical routes are indispensable for the algal cell, even for nonphotosynthetic pathogens. Remarkably, some of the Chlorellales‐shared plastid pathways (e.g. fatty acid, isoprenoid, and tetrapyrrole biosynthesis) are also retained in the colorless plastid of apicomplexans, which possess a secondary, red‐algal‐derived plastid (Fig. 1a) (De Koning & Keeling, 2006).

There are no complete nuclear genomes available yet for free‐living colorless chlorophytes, but such data will be important to investigate the evolutionary patterns following the loss of photosynthesis under an ecological scenario that is different from the parasitic/pathogenic lifestyle underlying the evolution of *Helicosporidium* and *Prototheca*. The analysis of transcriptomic data of diverse *Polytomella* species (Smith & Lee, 2014) and *Polytoma uvella* nuclear genome data (our unpublished data) has revealed numerous putative nuclear‐encoded, plastid‐targeted enzymes (Table 2) shared with *Helicosporidium* and *Prototheca* (Borza *et al*., 2005), suggesting that key nonphotosynthetic functions are maintained in both free‐living and parasitic/pathogenic nonphotosynthetic green algae.

**Table 2:** Putative nuclear‐encoded plastid targeted enzymes shared between different nonphotosynthetic green algae

	*Helic*	*Prowi*	*Poluv*	*Polpa*
Phenylalanine tyrosine and tryptophan biosynthesis				
Anthranilate phosphoribosyltransferase	**●**			**●**
3‐Phosphoshikimate 1‐carboxyvinyltransferase	**●**			
Aspartate aminotransferase, chloroplastic	**●**	**●**	**●**	**●**
Histidinol‐phosphate aminotransferase	**●**	**●**		
Shikimate kinase	**●**			**●**
3‐Deoxy‐7‐phosphoheptulonate synthase (aroF)	**●**	**●**	**●**	**●**
Anthranilate synthase component I	**●**			**●**
Anthranilate synthase component II	**●**			**●**
Tryptophan synthase alpha chain (trpA)	**●**		**●**	**●**
Tryptophan synthase beta chain (trpB)	**●**	**●**	**●**	
3‐Dehydroquinate synthase (aroB)	**●**	**●**		**●**
Chorismate synthase	**●**			**●**
Chorismate mutase	**●**			**●**
Arogenate/prephenate dehydratase (pheA)	**●**	**●**		**●**
3‐Dehydroquinate dehydratase/shikimate dehydrogenase	**●**			**●**
Arogenate dehydrogenase (NADP+), plant	**●**			**●**
Aspartate aminotransferase and glutamate/aspartate‐prephenate aminotransferase	**●**			**●**
Terpenoid backbone biosynthesis				
1‐Deoxy‐d‐xylulose‐5‐phosphate reductoisomerase	**●**			**●**
Protein‐S‐isoprenylcysteine *O*‐methyltransferase				
Acetyl‐CoA C‐acetyltransferase	**●**			**●**
Farnesyl diphosphate synthase (fps1)	**●**	**●**	**●**	**●**
4‐Diphosphocytidyl‐2‐C‐methyl‐d‐erythritol kinase	**●**			**●**
2‐C‐methyl‐d‐erythritol 4‐phosphate cytidylyltransferase				
Hydroxymethylglutaryl‐CoA synthase	**●**			
1‐Deoxy‐d‐xylulose‐5‐phosphate synthase	**●**			**●**
2‐C‐methyl‐d‐erythritol 2,4‐cyclodiphosphate synthase	**●**		**●**	**●**
Isopentenyl‐diphosphate delta‐isomerase	**●**			**●**
(E)‐4‐hydroxy‐3‐methylbut‐2‐enyl‐diphosphate synthase (gcpE)	**●**	**●**		**●**
4‐Hydroxy‐3‐methylbut‐2‐enyl diphosphate reductase	**●**	**●**	**●**	**●**
All‐trans‐nonaprenyl‐diphosphate synthase	**●**		**●**	**●**
Protein farnesyltransferase subunit beta	**●**			**●**
Protein farnesyltransferase/geranylgeranyltransferase type‐1 subunit alpha	**●**		**●**	**●**
Ditrans, polycis‐polyprenyl diphosphate synthase	**●**	**●**	**●**	**●**
Geranyl diphosphate synthase	**●**	**●**		**●**
Prenylcysteine alpha‐carboxyl methylesterase	**●**		**●**	**●**
Valine, leucine and isoleucine biosynthesis				
3‐Isopropylmalate dehydrogenase (leuB)	**●**			**●**
Ketol‐acid reductoisomerase (ilvC)	**●**	**●**	**●**	**●**
Branched‐chain amino acid aminotransferase	**●**		**●**	**●**
2‐Isopropylmalate synthase	**●**			**●**
Acetolactate synthase I/II/III large subunit (ilvI)	**●**	**●**	**●**	**●**
Acetolactate synthase I/III small subunit (ilvH)	**●**	**●**	**●**	**●**
Dihydroxy‐acid dehydratase	**●**		**●**	**●**
3‐Isopropylmalate/(R)‐2‐methylmalate dehydratase large subunit	**●**		**●**	**●**
3‐Isopropylmalate/(R)‐2‐methylmalate dehydratase small subunit				
Threonine dehydratase	**●**			**●**
Biosynthesis of unsaturated fatty acids				
3‐Oxoacyl‐[acyl‐carrier protein] reductase (fabG)	**●**			**●**
Acyl‐CoA oxidase	**●**		**●**	**●**
Stearoyl‐CoA desaturase (delta‐9 desaturase)				
Acyl‐[acyl‐carrier‐protein] desaturase	**●**	**●**	**●**	**●**
Acetyl‐CoA acyltransferase 1	**●**			**●**
Omega‐6 fatty acid desaturase (delta‐12 desaturase)	**●**	**●**	**●**	**●**
Very‐long‐chain enoyl‐CoA reductase	**●**			**●**
Very‐long‐chain (3R)‐3‐hydroxyacyl‐[acyl‐carrier protein] dehydratase	**●**		**●**	**●**
Porphyrin and chlorophyll metabolism				
Protochlorophyllide reductase	**●**			**●**
Coproporphyrinogen III oxidase	**●**		**●**	**●**
Oxygen‐dependent protoporphyrinogen oxidase	**●**			**●**
Heme oxygenase				
Ferritin heavy chain	**●**			**●**
Cob(I)alamin adenosyltransferase				
Uroporphyrinogen decarboxylase (hemE)	**●**	**●**	**●**	**●**
Porphobilinogen synthase (hemB)	**●**	**●**	**●**	**●**
Uroporphyrinogen‐III synthase (hemD)	**●**	**●**	**●**	**●**
Hydroxymethylbilane synthase	**●**		**●**	**●**
Cytochrome *c* heme‐lyase	**●**		**●**	**●**
Ferrochelatase (hemH)	**●**		**●**	**●**
Glutamate‐1‐semialdehyde 2,1‐aminomutase	**●**		**●**	**●**
Glutamyl‐tRNA synthetase	**●**		**●**	**●**
Protoheme IX farnesyltransferase	**●**		**●**	**●**
Cytochrome *c* oxidase assembly protein subunit 15	**●**		**●**	**●**
Glutamyl‐tRNA reductase	**●**		**●**	**●**
Oxygen‐independent coproporphyrinogen III oxidase	**●**		**●**	**●**
Starch and sucrose metabolism				
UDP glucose 6‐dehydrogenase	**●**			**●**
Starch phosphorylase	**●**		**●**	**●**
Sucrose‐phosphate synthase	**●**			
1,4‐Alpha‐glucan branching enzyme (glgB)	**●**	**●**	**●**	**●**
Starch synthase	**●**		**●**	**●**
4‐Alpha‐glucanotransferase				
1,3‐Beta‐glucan synthase	**●**			**●**
Hexokinase				
UTP‐glucose‐1‐phosphate uridylyltransferase				
Glucose‐1‐phosphate adenylyltransferase (glgC)	**●**	**●**	**●**	**●**
Trehalose 6‐phosphate phosphatase	**●**			**●**
Beta‐amylase				
Beta‐fructofuranosidase	**●**			**●**
Alpha‐trehalase	**●**	**●**	**●**	
Glucose‐6‐phosphate isomerase	**●**	**●**	**●**	**●**
Phosphoglucomutase (pgm)	**●**	**●**	**●**	**●**
Glycogen operon protein	**●**		**●**	**●**
UDP‐glucuronate 4‐epimerase	**●**			**●**
Trehalose 6‐phosphate synthase/phosphatase	**●**			**●**

*Helic*,* Helicosporidum* sp. ATCC 50920; *Prowi*,* Prototheca wickerhamii*;* Poluv*,* Polytoma uvella*;* Polpa*,* Polytomella parva*. Homologous sequences in *Prototheca* (Borza *et al*., 2005), *Polytomella* spp. (Smith & Lee, 2014) and *Polytoma uvella* (our unpublished data) genome‐scale sequence data were identified using BLASTP (*E*‐value ≤ 10^−30^) searches using *Helicosporidium* sp. protein models (Pombert *et al*., 2014) as query. Black circles indicate the presence of at least one homologous sequence.

Overall, we argue that genomic and transcriptomic studies of colorless green algae have the potential to greatly improve our understanding of photosynthesis and its evolutionary loss. The available data are largely skewed towards pathogenic/parasitic species and, thus, are impacted by the gene‐repertoire reduction associated with both the loss of photosynthesis and parasitism. Current transcriptomic data suggest that there is a certain degree of ‘convergence’ in the plastid protein repertoire among diverse colorless algae and land plants; however, the data available for colorless Chlamydomonadales are limited.

### Conclusions

Mixotrophy has been an important driving force in the loss of photosynthesis across diverse green algal lineages. The genera *Helicosporidium* and *Prototheca* are examples of loss of photosynthesis associated with the transition to parasitic/pathogenic lifestyles and sequencing additional nuclear genomes from *Helicosporidium* and *Prototheca* species will be important to understand the evolution of this trophic transition. For example, is the relative lack of genomic reduction in *Helicosporidium* sp. ATCC 50920 a common trend among pathogenic/parasitic trebouxiophytes? Unlike *Helicosporidium* and *Prototheca*,* Polytomella* and *Polytoma* are not believed to have gone through pathogenic/parasitic stages en route to losing photosynthesis. Thus, they should lack the genomic consequences typically associated with parasitism, such as high nucleotide substitution rates, gene loss, and reduced rates of recombination, and can provide a different perspective on our understanding of the loss of photosynthesis. Important insights could come from comparative genomic studies of *Polytomella* and *Polytoma* species and their close photosynthetic *Chlamydomonas* relatives.

For example, the colorless‐photosynthetic ‘sister taxa’ pairs *P. uvella*–*C. leiostraca* and *P. oviforme*–*C. chlamydogama* represent exceptional duos for investigating the consequences of the loss of photosynthesis in free‐living algae without the confounding effects associated with adopting a parasitic/pathogenic lifestyle. Our preliminary analyses of *Polytoma uvella* ptDNA sequence data reveal similar patterns of gene loss between the genomes of free‐living and parasitic chlorophytes. These similarities are notable given the different ecological scenarios that presumably drove the independent evolution toward heterotrophism, and suggest that the convergence in ptDNA gene content after the loss of photosynthesis has been shaped by similar constraints.

Other questions to be addressed concern the nuclear genomic complements of colorless green algae. For example, how do the gene collections of different *Polytoma* lineages and *Polytomella* species compare to the repertoire of their closely related photosynthetic taxa? Has the loss of photosynthesis caused expansions or contractions of particular gene families in colorless algae? Are there ‘unique’ genes, or even complete pathways, encoded in the nuclear genomes of the colorless species not present in those of their photosynthetic relatives? Has horizontal gene transfer had any role in the evolution of colorless Chlamydomonadales? How did the organelles of cyanobacterial origin recruited > 1 billion yr ago become essential for other cellular roles beyond the photosynthesis? Are there other biochemical and molecular functions, other than photosynthesis, critical for the establishment of primary plastids?

Finally, the study of the *Polytomella* and *Polytoma* nuclear genomes and plastid proteomes will be key to understand in detail the physiological roles of their colorless organelles. The very distinct evolutionary outcomes in the two free‐living chlamydomonadalean lineages that lost photosynthesis independently raise interesting questions, including: What mechanisms led to ptDNA loss in *Polytomella*? Did any ‘unique’ functions evolve independently in the colorless plastids of the different Chlamydomonadales? Answers to these basic questions will be critical to reveal the ecological and genomic process underlying the origin of these remarkable green algae that have evolved as ubiquitous free‐living organisms even after ‘the lights went out’.
